# Domestic trends in malaria research and development in China and its global influence

**DOI:** 10.1186/s40249-016-0222-x

**Published:** 2017-01-10

**Authors:** Yang-Mu Huang, Lu-Wen Shi, Rui She, Jing Bai, Shi-Yong Jiao, Yan Guo

**Affiliations:** 1 School of Public Health, Peking University Health Science Center, Xueyuan Road 38, Haidian District, Beijing, 100191 China; 2 School of Pharmaceutical Science, Peking University Health Science Center, Xueyuan Road 38, Haidian District, Beijing, 100191 China; 3 Department of Health Policy and Management, School of Public Health, Peking University Health Science Center, Xueyuan Road 38, Haidian District, Beijing, 100191 China; 4 Patent Examination Cooperation Center of the Patent Office, SIPO, Beijing, China

**Keywords:** Research and development, R&D, Malaria, Antimalarial, China

## Abstract

**Background:**

Though many countries, including China, are moving towards malaria elimination, malaria remains a major global health threat. Due to the spread of antimalarial drug resistance and the need for innovative medical products during the elimination phase, further research and development (R&D) of innovative tools in both epidemic and elimination areas is needed. This study aims to identify the trends and gaps in malaria R&D in China, and aims to offer suggestions on how China can be more effectively involved in global malaria R&D.

**Methods:**

Quantitative analysis was carried out by collecting data on Chinese malaria-related research programmes between 1985 and 2014, invention patents in China from 1985 to 2014, and articles published by Chinese researchers in PubMed and Chinese databases from 2005 to 2014. All data were screened and extracted for numerical analysis and were categorized into basic sciences, drug/drug resistance, immunology/vaccines, or diagnostics/detection for chronological and subgroup comparisons.

**Results:**

The number of malaria R&D activities have shown a trend of increase during the past 30 years, however these activities have fluctuated within the past few years. During the past 10 years, R&D on drug/drug resistance accounted for the highest percentages of research programmes (32.4%), articles (55.0% in PubMed and 50.6% in Chinese databases) and patents (45.5%). However, these R&D activities were mainly related to artemisinin. R&D on immunology/vaccines has been a continuous interest for China’s public entities, but the focus remains on basic science. R&D in the area of high-efficiency diagnostics has been rarely seen or reported in China.

**Conclusions:**

China has long been devoted to malaria R&D in multiple areas, including drugs, drug resistance, immunology and vaccines. R&D on diagnostics has received significantly less attention, however, it should also be an area where China can make a contribution. More focus on malaria R&D is needed, especially in the area of diagnostics, if China would like to contribute in a more significant way to global malaria control and elimination.

**Electronic supplementary material:**

The online version of this article (doi:10.1186/s40249-016-0222-x) contains supplementary material, which is available to authorized users.

## Multilingual abstracts

Please see Additional file 1 for translations of the abstract into the five official working languages of the United Nations.

## Background

Despite the development of multiple control efforts, malaria remains a global epidemic with a high burden of disease, particularly in impoverished regions [1]. Malaria has been contracted in over 96 countries inhabited by roughly 3.3 billion people, and approximately 214 million cases were recorded in 2014 [2]. Though many countries, including China, are moving towards malaria elimination, further research is still necessary, particularly in the areas of diagnostics, monitoring, evaluation and surveillance [3, 4]. Additionally, emerging challenges, such as artemisinin resistance and workforce migration could precipitate new epidemics [5].

A consensus has been reached on the importance of developing innovative tools to combat malaria and the new challenges it presents in both epidemic and elimination areas [6, 7]. For example, targeted treatments are needed for asymptomatic infections of low parasite densities in low transmission countries. Field-ready diagnostic tools for mass screening and surveillance are needed to monitor transmission reduction [7]. Novel drugs are also needed to prepare for the possibility of widespread artemisinin resistance, especially in vulnerable populations such as children and pregnant women.

The world expects developing countries to invest in R&D related to their own health concerns, such as tuberculosis and malaria [8]. The report of the Consultative Expert Working Group on R&D (CEWG) released by the World Health Organization (WHO) in 2012 proposed that all member states should cooperate and contribute additional funding to promote R&D into the diseases that disproportionately affect developing countries [9]. As one of the largest developing countries, China has the financial and technical capability to support medical R&D, and has shown potential in this field [10]. China has already made considerable progress in basic and operational research related to antimalarial technology. For example, China discovered artemisinin and its use in the treatment of malaria [11].

Continuous R&D is essential for China to combat malaria-related challenges, especially the multiple occurrences of drug resistance in the Greater Mekong Subregion [12]. Within this region, malaria transmission is particularly intense along the borders between Cambodia, Laos, Myanmar, Thailand and Vietnam [13]. Chloroquine-resistant falciparum malaria in China was first discovered in Yunnan in 1973, which led to the withdrawal of Chloroquine treatment for falciparum malaria in the 1980s [14]. Moreover, resistance to amodiaquine and sulfadoxine-pyrimethamine, as well as declining sensitivities to pyronaridine and mefloquine were also documented in the same regions [15, 16]. Although artemisinin combination therapies (ACTs) are recommended to delay resistance, drug efficacy studies have detected artemisinin resistance in the Greater Mekong Subregion, that is similar to the trend of Chloroquine resistance in the 1980s [2]. *P. falciparum* resistance to artemisinin is mostly restricted to the Greater Mekong Subregion, however, low efficiency of artemether-lumefantrine treatments among malaria patients has also been reported in South America [17]. Besides these, data from in vivo testing along the China-Myanmar border has indicated a markedly lower dihydroartemisinin-piperaquine sensitivity in *Plasmodium falciparum* from 2007 to 2013 [18]. The emergence of artemisinin resistance increases the risk of resistance to the partner medicines in the combination, which could be a global threat for malaria control and treatment as no back-up drug for ACTs is currently available [19].

Therefore, accelerated R&D into alternative malaria-related products is urgently needed [20]. However, due to the lack of market incentives and sustainable governmental funding, R&D progress on malarial products has been slow and the future of malaria R&D is now a concern [3]. Identifying the current situation and the obstacles of malaria R&D in China is essential for future R&D and is necessary to ensure that limited resources can be used to address the needs in vitally important areas. However, until recently, most published articles concerning malaria R&D in China have only analysed literature or articles, and have primarily focused on disease control, while less research has studied the overall malaria R&D situation in China [4, 21].

In this study, data on malaria R&D activities, including research programmes, articles, and invention patents, were collected and analysed to illustrate the situation of malaria R&D activities on basic science, human drugs, vaccines, and diagnostics in China. Chronological and subgroup comparisons were drawn to better demonstrate trends and China’s potential future contributions. The authors hope this research will better facilitate domestic elimination and prompt China to be more effectively involved in global malaria R&D and elimination.

## Methods

### Data sources and extraction

#### Research articles

A systematic search of articles published between January 1^st^, 2005 and December 31^st^, 2014 was conducted using PubMed, China National Knowledge Infrastructure (CNKI), and Wanfang databases to identify malaria R&D studies done by researchers in China. We used the following search criteria: *((“Malaria”) and ((“Vaccines, Malarial” or “Malaria Vaccines”) or (“Drugs, Antimalarial” or “Antimalarials”) or (“Diagnosis” or ““Malaria/diagnosis””)) and (“development” or “research”) and (“China”) and (“2005/01/01”[PDAT] : “2014/12/31”[PDAT])*. The excluded articles for this study were categorized as follows: review, education, software, comment, health promotion and commercial-related topics. The inclusion criteria were as follows: 1) original studies; 2) conducted in China; 3) closely related to the research topic. Two researchers screened the article titles and abstracts independently, and all abstracts that met the inclusion criteria were confirmed by a third researcher. Full articles were obtained for further exclusion if needed. All the selected articles were placed into four categories - basic sciences, drugs/drug resistance, immunology/vaccines, and diagnostics/detection.

#### Research programmes

National Nature Science Foundation of China (NSFC) is the main channel used by the Chinese government to support scientific research [22]. Malaria R&D programmes applied for and funded between 1985 and 2014 were obtained from the NSFC Internet-based Science Information system, using keywords “malaria” and “*Plasmodium*.” Two researchers excluded research on epidemiology/control and vector study separately. After reaching a consensus, they jointly placed the remaining research into the same four categories as used to classify the research articles.

#### Patents

Invention patents were obtained from China’s State Intellectual Property Office database, which included “Vaccines” or “Drugs” or “Diagnosis” and “Malaria” in the body of the text. Patents were mainly selected from subsectors A61 (Medical or Veterinary Science; Hygiene) and C12 (Biochemistry; microbiology; enzymology; mutation or genetic engineering) based on the International Patent Classification system. All patents were filed between 1985 and 2014 with rights having been granted by the end of 2012. The bibliographic information and date of each patent were obtained. After reading the information, two researchers excluded patents unrelated to malaria R&D and categorized the remaining patents into at least one of the three categories – drugs/drug resistance, immunology/vaccines, or diagnostics/detection.

### Data analysis

Since all data were organized in the Excel format (.xls), they were processed using Excel 2007 (Microsoft, WA, USA) filters. Excel was also used for index analysis, calculation, and visualization.

## Results

### General information

**Fig. 1:**
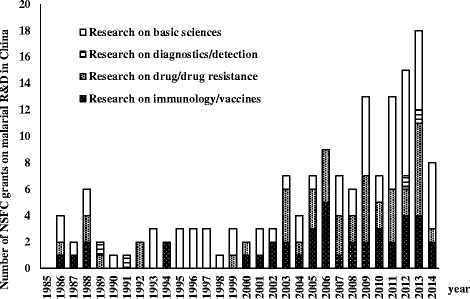
The number of research programmes granted by the National Nature Science Foundation of China (NSFC) related to R&D of malarial medical product between 1985 and 2014. No granted research was found in 1985 according to the NSFC database using the criteria

**Fig. 2:**
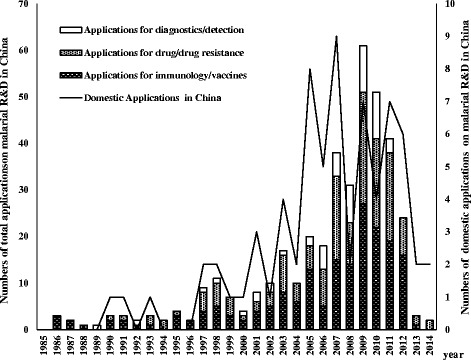
The number of total applications related to R&D of malarial medical product in China and the number of its domestic applications between 1985 and 2014. No applications were seen in 1985 under our research criteria

**Fig. 3:**
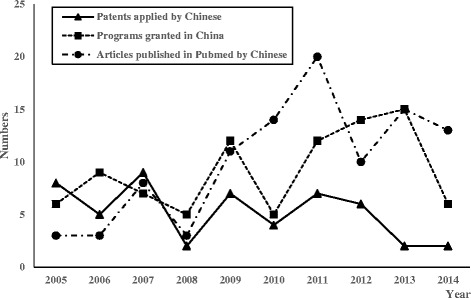
The numbers of research programmes granted by National Nature Science Foundation of China, articles published in PubMed by Chinese authors, and patents applied by Chinese related to R&D of malarial medical product between 2005 and 2014

In total, 100 (out of 322) English articles and 561 (out of 3 705) Chinese articles, 158 (out of 351) research programmes, 286 (selected from 347) patent applications and 82 (selected from 143) granted patents were analysed after the screening. The number of Chinese research programmes and patent applications related to malaria R&D showed a tendency of increase during the past 30 years (Figs. 1 and 2). The number of patent applications in China that met the study criteria rose dramatically from 21 during the first decade (from 1985 to 1994) to 289 during the third decade (from 2005 to 2014); while during the same intervals, domestic applications also rose tremendously from 3 to 58 (Fig. 2, Table 1). Similarly, 65.2% of research programmes were conducted during the most recent decade, while only 23 (14.6%) programmes were conducted from 1985 to 1994. Though R&D activities rapidly increased after the 1980s, the activities performed by Chinese researchers fluctuated and showed a downward trend within the last 10 years (Fig. 3). Moreover, an imbalance between different categories, especially between drug and diagnostics, were detected in all the activities (Figs. 1 and 2).

**Table 1:** Malarial invention patents filed by domestic and foreign entities and its grant ratios^a^

Fields	1985–1994						1995–2004						2005–2014					
	Domestic			Foreign			Domestic			Foreign			Domestic			Foreign		
	All	Public	Private	All	Public	Private	All	Public	Private	All	Public	Private	All	Public	Private	All	Public	Private
Vaccines	0 (0)	0 (0)	0 (0)	12 (8.3%)	1 (0)	11 (9.1%)	4 (75.0%)	3 (66.6%)	1 (100%)	38 (42.1%)	13 (30.8%)	25 (48.0%)	21 (57.1%)	15 (66.7%)	6 (33.3%)	111 (12.6%)	29 (13.8%)	82 (12.2%)
Drugs	3 (33.3%)	1 (100%)	2 (0)	3 (33.3%)	0 (0)	3 (33.3%)	11 (81.8%)	3 (100%)	8 (75.0%)	21 (42.9%)	5 (60.0%)	16 (37.5%)	34 (38.2%)	9 (55.6%)	25 (32.0%)	80 (11.3%)	17 (11.8%)	63 (11.1%)
Diagnostics	0 (0)	0 (0)	0 (0)	3 (33.3%)	1 (100%)	2 (0)	1 (100%)	0 (0)	1 (100%)	7 (57.1%)	2 (100%)	5 (40.0%)	3 (33.3%)	2 (0)	1 (100%)	40 (15.0%)	9 (22.2%)	31 (6.3%)
Total	3 (33.3%)	1 (100%)	2 (0)	18 (16.7%)	2 (50%)	16 (12.5%)	16 (81.3%)	6 (83.3%)	10 (80.0%)	66 (43.9%)	20 (45.0%)	46 (43.5%)	58 (43.5%)	26 (57.7%)	32 (60.0%)	231 (12.6%)	55 (14.5%)	176 (10.8%)

Grant ratios are reported in parentheses. Patents that were documented to be used in multiple areas, were counted repetitively so as to fully illustrate the R&D activities in certain areas

^a^The field is classified on the basis of international patent classification (IPC) taxonomy. Universities and research institutes are categorized as public applicants, while companies and individuals as private applicants

### R&D on drugs/drug resistance

R&D on drugs/drug resistance accounted for the highest proportion of research programmes (32.4%), articles published in Chinese (55.0% in PubMed and 50.6% in Chinese databases) and patent applications (45.5%) during the past decade. Compared to antimalarial drug R&D activities during the first decade, the number of activities within the past decade increased over 5.5 times (from 6 to 33 programmes) and 26 times (from 3 to 80 applications) in research programmes and patent application, respectively. Chinese applicants filed more patent applications than applicants outside China on antimalarial drugs. These were mainly filed for by private entities (Table 1).

Synthesis and pharmacodynamics of artemisinin and its derivatives were the main focus in China. The number of R&D activities related to artemisinin was the largest, with 48.5% (16/33) programmes and 61.8% (21/34) patent applications. Among 284 articles from Chinese databases and 55 written by Chinese researchers from PubMed, preclinical drug studies accounted for the highest proportion (57.5%), followed by 17.4% on clinical studies, 17.1% on drug resistance and susceptibility, 6.5% on drug safety and adverse effects, and 1.5% on other related subjects such as Glucose-6-phosphate dehydrogenase (G6PD) deficiency.

### R&D on immunology/vaccines

R&D on immunology/vaccines mainly focused on immunology and vaccine development (26 from PubMed and 80 from Chinese databases) rather than global antigenic polymorphism (3 from PubMed and 8 from Chinese databases). Patent applicants outside China showed consistently higher interest in this area compared to Chinese applicants (Table 1). Though only 27.5% of programmes and 17.7% of articles from China were related to R&D on immunology/vaccines during the past decade, China’s top public entities have continuously focused on this area. In China, vaccine development mainly focused on multi-epitope vaccine Malaria Random Constructed Antigen-1 (M.RCAg-1), screening of transmission blocking vaccine (TBV) antigen, and efficacy testing of PfCP2.9.

### R&D on diagnostics/detection

Globally, R&D on diagnostics and detection has mainly focused on diagnostic tools (78.9%) rather than biomarkers or detection of low parasitemia. R&D on serology-based assays and molecular-based approaches, such as rapid diagnostic tests (RDTs) and loop-mediated isothermal amplification (LAMP), have opened up new avenues for major improvements in malaria diagnostics. However, few R&D activities related to diagnostics were detected in China. Among them even fewer were related to novel approaches like LAMP (Figs. 1 and 2). The number of Chinese domestic patent applications did not show a significant increase during the past 30 years, while an increase was observed in foreign private entities during the past 10 years (Table 1).

### Research on basic science

In total, 110 (16.6%) articles written by Chinese researchers and 29 (31.9%) NSFC-funded programmes were related to basic science between 2005 and 2014. Genetics and development of *Plasmodium* was observed to be China’s main focus. This accounted for 48.2% of articles and 75.9% of NSFC programmes in the category of basic science. China’s emphasis on basic science, especially on *Plasmodium*, has proven to be a solid foundation and a great advantage for China in accelerating R&D on malaria drugs, vaccines, and diagnostics.

## Discussion

In this study, quantitative analysis was carried out to identify trends and potential gaps in China’s malaria R&D. Results showed that the number of malaria R&D programmes and patents increased tremendously during the past three decades. However, these activities have fluctuated within the past 3 years. This tendency could potentially be associated with China’s progress in combating malaria, and the notable shift of focus from communicable diseases to non-communicable diseases [23]. Our results indicated imbalanced malaria-related R&D activities among different categories in China, with most reported activities focused on antimalarial drugs and fewer related to diagnostics.

It is widely accepted that the R&D on malaria drugs, vaccines, and diagnostics is not only essential for epidemic areas, but also crucial for countries moving towards elimination [24, 25]. Indeed, significant R&D achievements have been made during the past few decades, such as ACTs, RDTs, and the RTS,S/AS01 malaria vaccine [4, 26]. The completion of genome sequences and stage-specific transcriptomes of the intraerythrocytic developmental cycle of *Plasmodium falciparum* and *P. vivax* may also lead to the discovery of new drugs and accelerate vaccine development [27–29]. However, most of these contributions were made by developed countries and foundations [20]. The world is expecting developing countries such as China to take on more research responsibilities, including technical and financial support [8, 9]. This study shows that China has long been devoted to malaria R&D on drugs/drug resistance, basic science, as well as immunology/vaccines, and may have the foundation to contribute to world malaria R&D.

China is expected to continue its R&D activities on malarial drugs, especially after winning the Nobel Prize for the discovery of artemisinin [30]. This research used the NSFC programme to illustrate the advantages and potential interests in malaria R&D, because of NSFC’s unique unlimited-topic application and selective peer-review system [22]. The high proportion (and increasing number) of NSFC-funded programmes and institutes interested in drugs strongly suggests that malaria researchers in China are primarily interested in antimalarial drugs. Furthermore, R&D on antimalarial treatments should continue to thrive due to China’s long history of traditional medicine. Indeed, preclinical studies on Chinese traditional medicine and new drug candidates for antimalarial use were seen in the R&D activities. However, R&D related to artemisinin and ACTs are still the main focus in China, which could impede China’s progress in new drug development.

Based on past experience with the spread of resistance to chloroquine and sulfadoxine-pyrimethamine, it is feared that artemisinin resistance will become widespread within a decade [31]. Though ACTs are expected to delay resistance, an acceleration of R&D activities for alternative antimalarial drugs is urgently needed [20]. Luckily, there is a pipeline of more than 10 new antimalarial drugs in various stages, such as the new antimalarial compound OZ439 [32, 33]. Routine monitoring and surveillance, as recommended by the WHO Global Plan for Artemisinin Resistance Containment, should be strengthened [34]. Unfortunately, this study shows that artemisinin-based drugs are still the main focus for researchers in China [35]. Concerns have been raised about the sustainability of R&D on antimalarial drugs, especially in a post-artemisinin world.

The recent decrease in the number of NSFC programmes and patent applications related to antimalarial drugs may also be an area of concern. This decrease may be the result of falling national support for malaria R&D after the burden of the disease has decreased in China [24]. Though prophylactic chemotherapy was suggested in high-transmission settings, the increasing cases of imported malaria from Southeast Asian and African countries have resulted in the spread of antimalarial drug resistances in China [24]. In addition, though most R&D activities focused on *P. falciparum*, regions of China are showing a predominance of *P. vivax* [36]. *P. vivax* is more difficult to control and eliminate than *P. falciparum* because of its tendency to relapse after resolution of the primary infection [3]. However, first-line therapies for the radical cure of *vivax* malaria - chloroquine and primaquine - have not changed in 60 years [37]. According to a systematic review of the resistance of *P. vivax* to chloroquine, chloroquine-resistant *P. vivax* is now present across most *vivax*-endemic regions [38]. All of the above challenges indicate that continued R&D activities are needed to achieve real malaria elimination.

Vaccines are considered amongst the most important modalities for potential prevention and reduction of malaria transmission [6]. However, the complexity of the *P. falciparum* life cycle, antigen polymorphism and lack of understanding for the protective immune mechanism have made vaccine development a daunting task [39]. Despite RTS,S - the only vaccine currently in Phase 3 clinical trials, there is no licensed malaria vaccine [40]. In this study, patent applicants from other countries showed consistently higher interest in vaccines since 1985 than Chinese domestic applicants. Domestic comparisons also indicated less R&D interest in vaccine than antimalarial drugs in China. However, the research indicated that China might have more potential in this category, since China’s top public entities in malaria research have been continuously focused on R&D on immunology/vaccines along with related basic science studies. Recently, M.RCAg-1 developed by Chinese scientists, was proven efficient in a pilot scale and was identified for its immunogenicity [41]. Vaccine candidate PfCP2.9 from China is listed on the WHO malaria vaccine rainbow table. And many antigens such as Pvs25 and Pvs28 were screened and tested for the development of TBV [42]. R&D on immunology/vaccines might be more promising than antimalarial drugs in China, however, continuous governmental support is essential for this time-consuming and costly battle.

Though efforts were seen in multiple R&D areas, the gaps between China and global malaria R&D are quite obvious, especially in the field of diagnostics and detection, as also mentioned by other studies [4, 35]. New screening tools and better diagnostic tools are recommended to be higher priorities for operational R&D, especially for countries moving towards elimination [4]. Field-ready active testing for mass screening and sensitive diagnostic tools for asymptomatic patients are necessary for global malaria elimination [7]. A number of specific serology-based assays (FAST-ELISA, RDTs), molecular-based approaches (LAMP) and proteomic technology for the discovery of biomarkers have emerged, and have been proven feasible in field settings with limited technical resources [4]. However, few R&D activities related to diagnostics were detected in China by this study. Also, the existing studies mainly focused on PCR, ELISA or RDTs, rather than novel approaches like LAMP. Effective biomarkers are also urgently needed to distinguish imported cases from local cases during the elimination stage. Unfortunately, in agreement with Zheng’s study, only a few activities related to this area were detected [35].

The current national strategy of China forcefully pushes for independent drug innovations to gain a competitive edge in the global market. From the authors’ point of view, China should encourage more international R&D cooperation, especially when it comes to infectious diseases or diseases mainly affecting developing countries. It is suggested by a US researcher that Sino-US scientific collaborations could be a powerful and winning combination to fight against globally neglected tropical diseases, and this collaboration should also include African scientists [43]. Beyond the potential benefits to China’s reputation, it is worth reflecting on broader benefits; more experience in international cooperation and better understanding of global R&D policy and practice. All of these will not only benefit China’s future involvement in global R&D, but also inject new forces to improve R&D on antimalarial products.

Though this was the first research that studied multiple malaria-related R&D activities in China, it is still subject to several limitations. Firstly, although vector control is an essential component for reduction of malaria transmission [44], research related to entomology and insecticides are not included here since human medical products are the focus of this study. Secondly, this study did not deliberately demarcate medical products for *P. vivax* and *P. falciparum* during data analysis, which otherwise might provide additional R&D suggestions. Also, though PubMed, Wanfang and CNKI were consciously selected in the hope of collecting all R&D related articles, these databases might not include some articles, not to mention research that remains unpublished or had negative results.

## Conclusions

This study indicated that China has long been devoted to malaria R&D on drug/drug resistance, basic science, and immunology/vaccines, while R&D related to diagnostics and detection of malaria is a major research gap for China. Continuous focus and international cooperation on malaria R&D is recommended for China to achieve domestic elimination and contribute to global malaria control and elimination.

## Additional file

Additional file 1: Multilingual abstracts in the five official working languages of the United Nations. (PDF 467 kb)

## Abbreviations

ACTs: Artemisinin combination therapies
CEWG: Consultative Expert Working Group
CNKI: China National Knowledge Infrastructure
G6PD: Glucose-6-phosphate dehydrogenase
LAMP: Loop-mediated isothermal amplification
M.RCAg-1: Malaria Random Constructed Antigen-1
R&D: Research and development
RDTs: Rapid diagnostic tests
TBV: Transmission blocking vaccine
WHO: World Health Organization
