# Supplementary material for: Domestic trends in malaria research and development in China and its global influence
Source: Infect Dis Poverty. 2017 Jan 10;6:4. doi: 10.1186/s40249-016-0222-x (PMC5223349; doi:10.1186/s40249-016-0222-x)
Supplement: Additional file 1: — Multilingual abstracts in the five official working languages of the United Nations. (PDF 467 kb) [file 40249_2016_222_MOESM1_ESM.pdf]

Translation of the abstract into the five official working languages of the United Nations

الاتجاهات المحلية في تطوير بحوث الملاريا في الصين وتأثيرها على المستوى العالمي

يانج مو وانج، لو-وين شي، روي شى، جينج باي، شي-يونج جياو، يان جوو.

ملخص
الخلفية: بالرغم من أن العديد من الدول بما فيها الصين تتجه نحو القضاء على الملاريا، إلا أن هذا المرض لا يزال يمثل تهديدًا صحيًا عالميًا بارزًا. ونظرًا لإنتشار الملاريا المقاومة للعقاقير الطبية والحاجة إلى تصنيع منتجات طبية مبتكرة أثناء فترة القضاء على المرض، فثمة حاجة إلى إجراء المزيد من الأبحاث والتطوير لأدوات مبتكرة في مجالي الوباء والقضاء على المرض. ولذا تهدف هذه الدراسة إلى تحديد الاتجاهات والفجوات في أبحات تطوير مرض الملاريا في الصين وتهدف إلى طرح مقترحات بشأن كيفية مشاركة الصين على نحوٍ أكثر فاعلية في أبحثت وتطوير الملاريا على المستوي العالمي.
الوسائل: تم إجراء تحليل كمي بواسطة تجميع البيانات حول برامج الأبحاث الصينية المتصلة بالملاريا بين عام ١٩٨٥ وعام ٢٠١٤، وبراءات الاختراع في الصين بين عام ١٩٨٥ وعام ٢٠١٤، والمقالات التي نشرها باحثون صينيون في دورية PubMed وقواعد البيانات الصينية من عام ٢٠٠٥ إلى عام ٢٠١٤. ولقد تم تحليل كافة البيانات واستخراجها لإجراء تحليل رقمي وتم تصنيفها تحت علوم أساسية، أو عقاقير/مقاومة العقاقير، أو علم المناعة/ اللقاح، أو التشخيص/ الاكتشاف لإجراء مقارنات زمنية وللمجموعات الفرعية.
النتائج: لقد أظهرت أنشطة أبحاث وتطوير الملاريا اتجاه مرتفع خلال الثلاثين عامًا السابقة، إلا أن تلك الأنشطة قد تأرجحت خلال الأعوام القليلية السابقة. وخلال العشرة أعوام السابقة، مثلت الأبحاث والتطوير الخاصة بالعقاقير/ مقاومة العقاقير أعلى نسب في برامج البحث (٣٢,٤٪)، والمقالات (٥٥,٠٪ في PubMed و٥٠,٦٪ في قواعد البيانات الصينية) وبراءات الاختراع (٤٥,٥٪). ومع ذلك، فلقد اتصلت أنشطة الأبحاث والتطوير تلك بالأرتيمسينين بالدرجة الأولى، فإن الأبحاث والتطوير الخاصة بعلم المناعة/اللقاح لطالما كان محط اهتمام المؤسسات العامة في الصين غير أن التركيز لا يزال ينصب على العلوم الأساسية. اما الأبحاث والتطوير في مجال التشخيص عالي الكفاءة فنادرًا ما تم ملاحظته أو تحديده في الصين.
الاستنتاجات: لقد كانت الصين حريصة منذ وقت طويل على إجراء الأبحاث والتطوير الخاصة بالملاريا في مجالات متعددة، بما فيها العقاقير الطبية والمقاومة للعقاقير وعلم المناعة واللقاح. ولقد تلقت الأبحاث والتطوير فيما يتعلق بالتشخيص عناية أقل على نحوٍ ملحوظ. غير أن الأبحاث والتطوير يجب أن تكون مجالًا يجب أن تسهم الصين فيه. كما يجب أن ينصب التركيز أكثر على الأبحاث والتطوير في الملاريا ولاسيما في مجال التشخيص وهذا إذا كانت ترغب الصين في الاسهام على نحوٍ أكثر تأثيرًا في مكافحة مرض الملاريا والقضاء عليه.

Translated from English version into Arabic by Norhan Mahmo, through

TRANSLATORS
WITHOUT BORDERS

中国疟疾医药产品研究与开发的国内趋势及国际影响

黄旸木，史录文，佘睿，白婧，焦士勇，郭岩

摘要
引言：虽然中国等许多国家正迈入疟疾消除阶段，疟疾仍然是一个全球重大健康威胁。基于耐药性的蔓延和消除阶段对新型医疗产品的需求，疟疾的流行和消除地区都有需求进一步研

究和开发（研发）新型抗疟医药产品。本研究旨在分析中国抗疟医药产品研发的趋势和差距，为中国如何更有效地参与全球疟疾研发提供建议。
方法：采用定量分析方法，收集 1985 年至 2014 年中国开展的疟疾相关研究项目，1985 年至 2014 年在中国申请和授权的相关发明专利，及 2005 年至 2014 年在 PubMed 和中文数据库中收录的由中国研究人员发表的相关文章。经过筛选，所有资料被纳入基础科学类、药物/耐药性类、免疫/疫苗类、诊断/检测类，用于时间和分组比较。
结果：疟疾相关研发活动数量在过去 30 年呈增加趋势，但近几年略有波动。近十年，与药物/耐药性有关的研发在研究项目（32.4%）、文章（占 PubMed 的 55.0%，占中文数据库的 50.6%）和专利（45.5%）中占最高比例，但主要与青蒿素有关。中国公共机构持续关注免疫/疫苗相关研发，但以基础研究为主。高效率诊断产品的相关研发在中国少有报道。
结论：中国长期开展与疟疾药物、耐药性、免疫和疫苗相关的多领域研发。诊断相关研发活动较少，但仍是中国可能发挥作用的一大领域。中国如果希望在全球疟疾防控和消除中作出更大贡献，应该更多关注疟疾相关医药产品研发，特别是诊断相关研发。

Translated from English version into Chinese by Yang-Mu Huang

Tendances domestiques en recherche et développement sur la lutte contre le paludisme en Chine et son influence mondiale

Yang-Mu Huang, Lu-Wen Shi, Rui She, Jing Bai, Shi-Yong Jiao, Yan Guo

Résumé

Contexte: À un moment où de nombreux pays, y compris la Chine, avancent vers une éradication du paludisme, cette maladie reste l'une des principales menaces pour la santé à l'échelle mondiale. Face à la propagation de la résistance aux anti-paludéens et aux besoins en produits médicaux novateurs pendant la phase d'éradication, la recherche et le développement (R&D) d'instruments novateurs avancés en matières d'épidémie et d'éradication sont une nécessité. Cette étude vise donc à identifier les tendances et les lacunes en R&D sur la lutte contre le paludisme en Chine, et cherche aussi à apporter des suggestions visant à rendre la Chine plus efficace et plus active dans la recherche et le développement sur la lutte contre le paludisme dans le monde.
Méthodologie: Une analyses quantitative a été faite sur la base de données collectées à partir des programmes de recherche sur le paludisme et des brevets d'inventions en Chine de 1985 à 2014, ainsi qu'à partir d'articles publiés par des chercheurs chinois dans PubMed et dans les bases de données chinoises de 2005 à 2014. Toutes ces données ont été passées au crible puis extraites pour une analyse quantitative. Elles ont ensuite été classifiées par science élémentaire, par médicaments/résistance aux médicaments, par immunologie/vaccins, ou par diagnostique/détection afin de mener une étude comparative des chronologies et des sous-groupes.
Résultats: La tendance du nombre d'activités en recherche et développement sur la lutte contre le paludisme était en hausse au cours des 30 dernières années, activités qui ont toutefois connu des fluctuations au cours des dernières années. Par ailleurs, au cours des 10 dernières années, on note que la recherche et le développement de médicaments/la résistance aux médicaments constituent les domaines sur lesquels se sont le plus appesantis les programmes de recherche, soit (32,4%), pour les articles (55,0% dans PubMed et 50,6% dans les bases de données chinoises), et (45,5%) pour les

brevets. Il n'en demeure pas moins que ces activités de R&D n'étaient concentrées que sur l'artemisinin. Les organismes publics de Chine, à l’heure actuelle continuent de porter un intérêt à la recherche et au développement sur l'immunologie/les vaccins, mais l'accent demeure sur leur science élémentaire. Des cas de recherche et de développement de diagnostiques hautement efficaces ont donc rarement été vus ou évoqués en Chine.

Conclusions: La Chine s'est longtemps dévouée à la recherche et au développement dans plusieurs domaines de la lutte contre le paludisme, notamment sur les médicaments, la résistance aux médicaments, l'immunologie et les vaccins. Par contre, la recherche et le développement de diagnostiques a reçu beaucoup moins d'attention, alors que c'est un secteur où la Chine peut apporter sa contribution. Ainsi, au cas où la Chine voudrait contribuer d'une manière plus importante au contrôle et à l'éradication du paludisme dans le monde, elle devra accorder encore plus d'attention à la recherche et au développement de moyens de lutte contre le paludisme en général, et au développement de diagnostiques en particulier.

Translated from English version into French by simonyetna, through

TRANSLATORS
WITHOUT BORDERS

Научно-практические исследования малярии в Китае: национальные тенденции и их глобальное влияние

Янму Хуан, Лувэнь Ши, Жуй Шэ, Цзин Бай, Шиюн Цзяо, Янь Го

Аннотация

Обоснование. Малярия остается одной из основных угроз здоровью людей по всему миру, несмотря на усилия многих стран, в том числе Китая, направленные на ее элиминацию. В связи с распространением устойчивости к противомалярийным препаратам и потребностью в инновационных лекарственных средствах во время этапа элиминации, необходимо дальнейшее проведение научно-практических исследований (R&D) инновационных инструментов для прекращения эпидемий и элиминации малярии. Цель этого исследования — определить тенденции и пробелы в научно-практической работе в области малярии в Китае. Кроме того, в нем высказывается ряд предложений по более эффективному вовлечению Китая в глобальные научно-практические исследования малярии.

Методы. Количественный анализ был проведен путем сбора данных, касающихся изучения малярии. А именно: исследовательские программы в Китае (1985–2014), выданные в Китае патенты на изобретения (1985–2014), а также статьи, написанные китайскими исследователями и опубликованные в базе данных PubMed и китайских базах данных (2005–2014). Данные были проверены и извлечены для численного анализа, затем распределены для хронологического сравнения и сравнения среди подгрупп по следующим категориям: основные медицинские дисциплины, лекарственные препараты и устойчивость к ним, иммунология и вакцинация, диагностика и выявление.

Результаты.В последние 30 лет наблюдается увеличение числа научно-практических работ в области малярии, но стоит обратить внимание тематические различия. За последние 10 лет научно-практические работы, посвященные лекарственным препаратам и устойчивости к ним, получили наибольшее распространение в исследовательских программах (32,4%), публикациях (55,0% в базе PubMed и 50.,6% в китайских базах публикаций) Впрочем, большинство этих работ касались артемизинина. У государственных учреждений Китая постоянный интерес вызывают научно-практические исследования иммунологии и вакцинации, однако их главное внимание по-прежнему обращено на основные медицинские дисциплины. Высокоэффективная диагностика остается редким направлением научно-практических исследований в Китае.

Выводы.Уже долгое время Китай прилагает значительные усилия в области научно-практические исследований малярии, акцентируя внимание на лекарственных препаратах и устойчивости к ним, иммунологии и вакцинации. Значительное меньшее внимание в научно-исследовательской деятельности было уделено диагностике. Китайским ученым стоит развивать направление. Чтобы сделать существенный вклад в сокращение и элиминацию малярии в глобальном масштабе, необходимо обратить более пристальное внимание на научно-практические исследования малярии и особенно вопросы диагностики.

Translated from English version into Russian by Aliaksandra Baravikova, through

TRANSLATORS
WITHOUT BORDERS

Tendencias nacionales en la investigación y desarrollo en la lucha contra la malaria en China y su influencia mundial

Yang-Mu Huang, Lu-Wen Shi, Rui She, Jing Bai, Shi-Yong Jiao, Yan Guo

Resumen

Contextualización: aunque muchos países, entre los que se incluye China, avanzan hacia la eliminación de la malaria, ésta sigue siendo una amenaza importante para la salud mundial. Debido a la propagación de la resistencia a los medicamentos contra la malaria y a la necesidad de contar con productos médicos innovadores en la fase de eliminación, es preciso que se investiguen y desarrollen (I+D) herramientas innovadoras tanto en las zonas epidémicas como en las que están en fase de eliminación. El objetivo de este estudio es identificar las tendencias y las deficiencias en la I+D sobre la malaria en China para ofrecer propuestas sobre cómo este país puede participar de manera más efectiva en la I+D internacional sobre la malaria.

Métodos: se llevó a cabo un análisis cuantitativo recopilando datos sobre los programas chinos relacionados con la malaria entre 1985 y 2014, las patentes de invención en China desde 1985 a 2014 y los artículos publicados por investigadores chinos en PubMed y bases de datos chinas desde 2005 a 2014. Se examinaron todos esos datos, se extrajeron para realizar un análisis numérico y se clasificaron en ciencias básicas, fármacos/ farmacorresistencia, inmunología/ vacunas o

diagnóstico/ detección para realizar comparaciones cronológicas y de subgrupos.
Resultados: el número de actividades de I+D muestra una tendencia al alza en los pasados 30 años; sin embargo, estas actividades han ido fluctuando. En los últimos 10 años, la I+D sobre fármacos/ farmacorresistencia representaba el porcentaje más alto en programas de investigación (32,4%), en artículos (55,0%) en PubMed y 50,6% en bases de datos chinas) y en patentes (45,5%). No obstante, esas actividades de I+D estaban principalmente relacionadas con la artemisinina. La I+D sobre inmunología/ vacunas es un continuo interés para los organismos públicos chinos, pero se centra en la ciencia básica. Es poco común encontrar I+D en el campo de los métodos diagnósticos de alta eficacia en China.
Conclusiones: hace tiempo que China se dedica a la I+D sobre malaria en múltiples aspectos entre los que se incluyen los fármacos, la farmacorresistencia, la inmunología y las vacunas. Se le ha prestado bastante menos atención a la I+D sobre métodos diagnósticos, aunque China también puede hacer aportes a este campo. Es necesario que China le dé más prioridad a la I+D sobre malaria, especialmente en cuanto a su diagnóstico, si quiere contribuir de un modo más significativo al control y la eliminación mundial de esta enfermedad.

Translated from English version into Spanish by Laura Rodríguez Manso, through

TRANSLATORS
WITHOUT BORDERS
